# Rhino-orbital cerebral mycosis: a case series of non-mucorales in COVID patients

**DOI:** 10.1099/acmi.0.000575.v4

**Published:** 2023-10-10

**Authors:** Rajashri Patil, Jyoti Ajagunde, Sameena Khan, Sriram Kannuri, Nageswari Gandham, Sahjid Mukhida

**Affiliations:** ^1^​ Department of Microbiology, Dr. D. Y. Patil Medical College, Hospital and Research Centre, Dr. D. Y. Patil Vidyapeeth, Pimpri, Pune 411018, Maharashtra, India

**Keywords:** mycosis, *Neurospora*, *Fusarium*, *Cladosporium*, post-COVID

## Abstract

**Introduction.:**

Rhino-Orbito-cerebral mycoses are not only caused by *Aspergillus* spp. and *Zygomycetes* spp. but also can be associated with other rare species such as *Neurospora* spp.*, Cladosporium* spp. and *Fusarium* spp. Mucormycosis is associated causatively with immunocompromised states, for example patients with comorbidities such as diabetes mellitus. Clinical symptoms of coronavirus disease (COVID) and mucormycosis in tandem are critical and relentless, frequently with no life-saving treatment.

**Case series.:**

We report three patients with COVID-19 infection, who during the course of treatment developed rhino-orbital-cerebral mycosis including oral cavity involvement. Rhinocerebral mycosis along with oral cavity involvement was diagnosed by radiological investigations and preliminary screening for fungal infection (KOH mount) in all three cases. Empirical treatment was started but patients did not respond to treatment. All patients died even after debridement and maxillectomy. On culture, rare species of fungi were isolated in all three cases which, with the help of a reference laboratory, were identified as *Neurospora*, *Cladosporium* and *Fusarium. Neurospora* is considered nonpathogenic to humans. *Cladosporium* is a dematiaceous fungus found in soil in all climates, associated with disseminated or cerebral infections; and *Fusarium*, though considered a saprophytic colonizer of skin and respiratory mucosa along with other bacteria, is a common cause of mycotic keratitis worldwide.

**Conclusion.:**

Immune system modifications due to COVID-19 with/without other risk factors can result in fungal co-infections that prove to be fatal for the patients. It is vital to be aware that COVID-19 patients, particularly those who are critically ill, may acquire secondary fungal infections and early detection is critical.

## Data Summary

No data was generated during this research or is required for the work to be reproduced.

## Introduction

Since 2019, the world has faced the scourge of severe acute respiratory syndrome coronavirus 2 (SARS-CoV-2). Rhino-orbital cerebral mycosis (ROCM) is a fatal opportunistic infection characterized by rapid progression and vascular invasion. Paltauf first identified rhinocerebral mycosis (RCM) in 1885 [1]. The most common causes of central nervous system (CNS) mycoses are zygomycosis and aspergillosis. Zygomycetes are part of the phylum Glomeromycota subphylum Mucoromycotina. *Rhizopus*, *Mucor*, *Rhizomucor* and *Absidia* are among the genera [2]. *Candida, Cryptococcus, Histoplasma, Coccidioides* and *Cladosporium* are other fungi that cause CNS mycosis [3].

ROCM is a rapidly progressive and fatal disease that begins after a patient inhales spores and it affects the lining of the nose and sinuses, the oral cavity and the eye sockets, and the CNS. The most common clinical manifestations of orbital involvement are headache, facial discomfort and fatigue. Symptoms increase rapidly and may be followed by ptosis, limited eye movement and periorbital cellulitis [4]. Visual impairment due to central retinal artery occlusion is also common. The most dangerous consequence of RCM is cerebral vasculitis [5]. The vascular invasion capacity of the fungus is primarily responsible for the arterial occlusion and extensive tissue necrosis. Various studies have shown the mortality rate in RCM to be 15–82 % [6].

Multiple resections, including maxillectomy with intravenous amphotericin B, combined with rigorous surgical management may provide a better prognosis and higher long-term survival [7]. Coronavirus disease 2019 (COVID-19) infection is associated with many forms of disease caused by fungal and bacterial co-infections [8]. We report three cases of COVID-19 with ROCM.

## Case report 1

A 66-year-old man presenting to the tertiary care hospital with shortness of breath and high-grade fever for the past 2 days tested positive for COVID-19 and was hospitalized on 6 May 2021. His SpO_2_ level was 80 % on admission and he was only able to maintain saturation with the help of non-invasive ventilation (NIV). He was monitored in the intensive care unit (ICU) and given remdesivir intravenously (IV) for 11 days. Methylprednisolone was given IV for 18 days. During his COVID-19 treatment, 12 days of injection dexamethasone was also administered. The patient began to complain of numbness, weakness and tingling all over the face (left side) 6–8 days after admission, with redness and tearing in the right eye for the last 1–2 days. The patient’s occupational history was that of a retired disabled worker who later worked as a fruit seller.

**Fig. 1.**
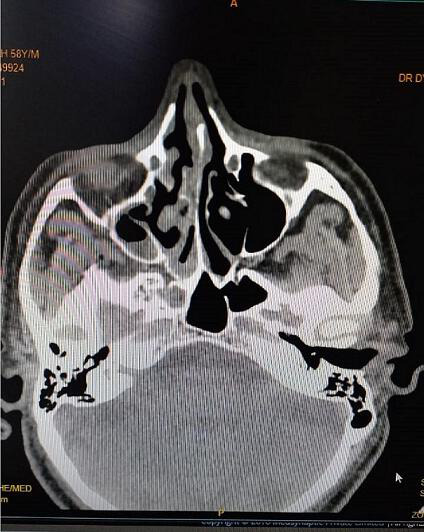
Case 1. CT PNS: the ethmoidal and maxillary nasal sinuses display an ill-defined heterogeneous soft tissue signal intensity of mucosal thickening.

**Fig. 2.**
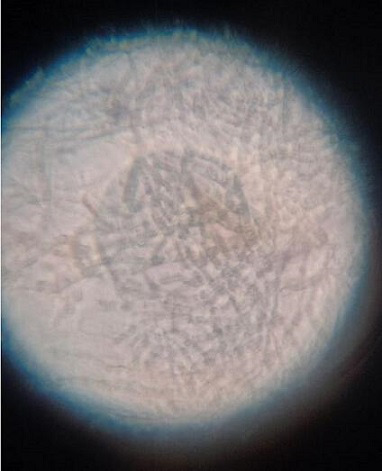
Case 1. KOH mount: fungal ball-type hyphae observed in a 10% KOH mount.

**Fig. 3.**
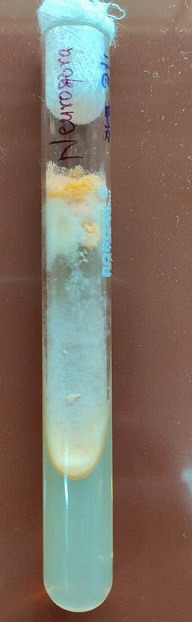
Case 1. *Neurospora* spp. growth on an SDA slant.

**Fig. 4.**
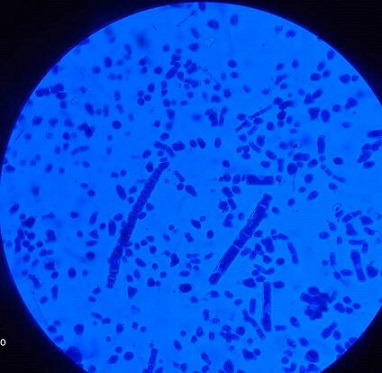
Case 1. LPCB mount: *Neurospora* spp. showing chains of arthroconidia.

The patient had type 2 diabetes mellitus (T2DM) for 8 years and hypertension for 5 years, and was inconsistent with treatment compliance. On computed tomography (CT) of the paranasal sinuses (PNS), the ethmoidal and maxillary nasal sinuses displayed an ill-defined heterogeneous soft tissue signal intensity of mucosal thickening (Fig. 1). According to test results, D-dimer was 790 ng ml^−1^, C-reactive protein (CRP) was 106 mg d^l−1^, Total Leukocytes Count (TLC) was 20500 mm^−3^, serum ferritin was 818 mg l^−1^, HbA_1_C was 10.8% and Lactate dehydrogenase (LDH) was 179 IU l^−1^. All of the parameters were abnormal. Based on all indications and symptoms, the patient was referred to undergo functional endoscopic sinus surgery (FESS). On the left side, FESS was performed on both the maxillary sinuses and the ethmoid sinus, some crusting was noted, and debrided tissue was sent to the pathology lab for histological investigation and to the microbiology lab for KOH mount and fungal culture. Histopathology revealed dense infiltration by inflammatory cells comprising predominantly lymphocytes, few plasma cells and foreign body giant cells in the epithelium and sub-epithelium with septate fungal hyphae having a broad base. In the KOH mount, a septate hyphae fungal ball was seen (Fig. 2). Within 2 days of culture, fungal growth showed aerial mycelium with an orange colour (Figs 3 and 4)

Conventional amphotericin B was initiated based on radiological and microbiological interpretation. Growth of *Neurospora* species was seen in culture on Sabouraud dextrose agar (SDA) plates at both 25 and 37 °C, 4–5 days after the material was received. His mouth cavity, including the maxillary and palate bones, was also implicated. A maxillectomy was performed, and the debrided tissue was sent for microbiological examination, which revealed the same type of organism in the KOH mount and culture. The patient’s condition did not improve, magnetic resonance imaging (MRI) of the brain and orbit revealed right orbital involvement and right intracranial haemorrhage, and the patient was scheduled for right eye exenteration. Following surgery, the patient was in the ICU for more than 40 days, and following surgery, the patient was on liposomal amphotericin B for up to 25 days but died 2 months later due to CNS involvement.

## Case report 2

A 63-year-old male patient on NIV was transferred from another hospital on 21 May 2021. At the time of admission, the patient complained of shortness of breath and right-sided facial and nasal discomfort. He had T2DM for 7 years and was on oral hypoglycaemics. He tested positive for COVID-19 within 14 days of hospitalization. For the first 7 days, the patient was quarantined at home, but his condition deteriorated, and he was taken to a government hospital. The chest severity score on high-resolution CT was 18 out of 25.

Laboratory investigation findings were D-dimer 4614 ng ml^−1^, CRP 78 mg dl^−1^, serum ferritin 927 mg l^−1^ and TLC 16900 mml^−3^. During his hospital stay, he had six injections of remdesivir 100 mg, methylprednisolone for 5 days and dexamethasone for 4 days, but his condition did not improve. The patient began complaining of severe headaches, and the treating physician suspected fungal sinusitis. The patient was sent to our hospital for further management. Because the patient’s health was worsening rapidly, he was admitted to the ICU with oxygen support.

**Fig. 5.**
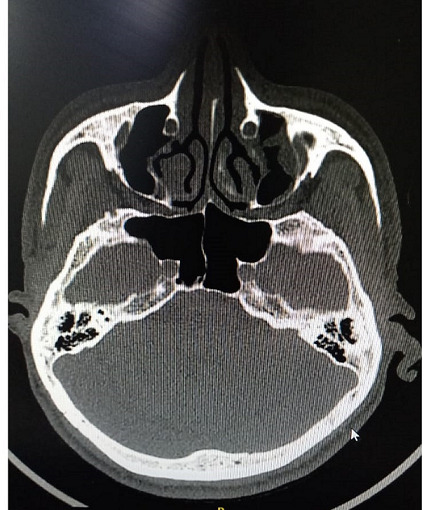
Case 2. CT PNS: mucosal thickening in the ethmoidal and maxillary nasal sinuses.

**Fig. 6.**
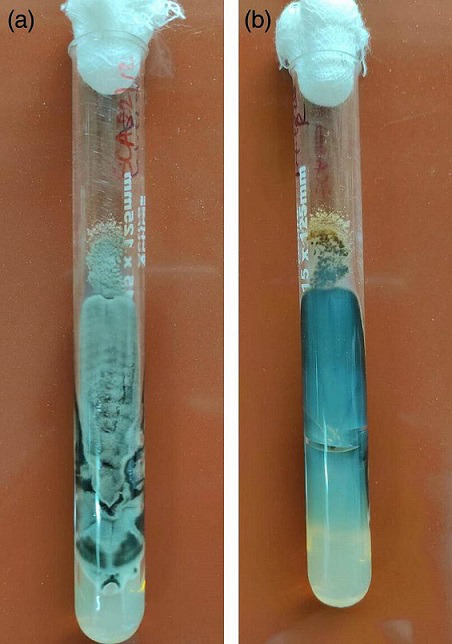
Case 2. *Cladosporium* species growth: (a) obverse and (b) reverse view.

CT PNS revealed mucosal thickening within the ethmoidal and maxillary nasal sinuses (Fig. 5). Because of a high level of suspicion, the patient was posted for FESS, and the debrided sample was submitted for histopathology, KOH and fungal culture. The KOH mount revealed septate-type fungal elements. The routine protocol for a positive fungal culture was the same as in Case 1. From the material, a *Cladosporium* species was isolated. A lactophenol cotton blue (LPCB) mount exhibited dark-pigmented conidia that are mostly in branching chains in olive-green to black colonies (Fig. 6). In this case, histopathology did not find any evidence of fungal hyphae.

The patient was treated empirically with liposomal amphotericin B, but showed no improvement. Because of the involvement of the oral cavity and palate bones, a partial maxillectomy was performed. The patient’s health deteriorated, and he ended up on ventilator support; his condition did not improve even after 15 days of hospitalization and ICU care, and he died.

## Case report 3

**Fig. 7.**
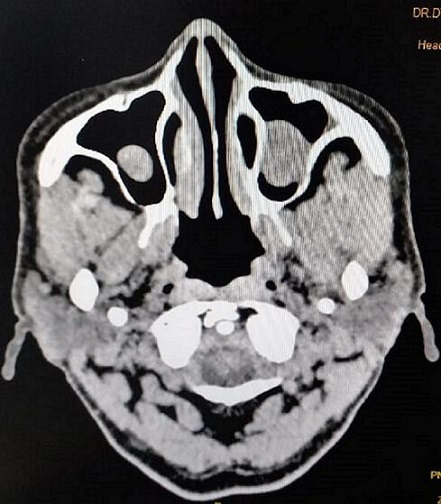
Case 3. CT PNS: mucosal thickening in the maxillary and ethmoidal sinuses.

A 50-year-old female patient presented to the hospital with shortness of breath, fever and unconsciousness on 25 May 2021. The patient tested positive for COVID-19 1 week previously. She had recently been diagnosed with T2DM and was on medication for hypertension for 2 years. She was immediately taken to the ICU for life-saving treatment. On admission, the patient’s oxygen saturation was 62 %. Subsequently, she were intubated and was on supported ventilation for 20 days. Laboratory investigation revealed D-dimer 1142 ng ml^−1^, CRP 54.7 mg dl^−1^, TLC 9800 mm^−1^, erythrocyte sedimentation rate 80 mm h^−1^, HbA_1_C 11.3 %, IL-6 18.51 pg ml^−1^ and LDH 478 IU l^−1^. All parameters were abnormal. During her hospital stay, the patient was given remdesivir 100 mg injection for 6 days and methylprednisolone 40 mg twice daily for 20 days. During her stay, she also received low molecular weight heparin and convalescent plasma. On MRI/CT of PNS and brain, mucosal thickening in the maxillary and ethmoidal sinuses was evident (Fig. 7). The otolaryngology consultant ordered a nasal swab (since FESS was not possible because the patient was on a ventilator) to rule out fungal aetiology and KOH for initial screening of fungal infection. We promptly notified the presence of fungal elements. The swab was subsequently inoculated onto SDA, and the positive fungal culture protocol was the same as in Case 1. A histopathological investigation was not sought, due to clinical judgement indicating a high likelihood of fungal infection.

**Fig. 8.**
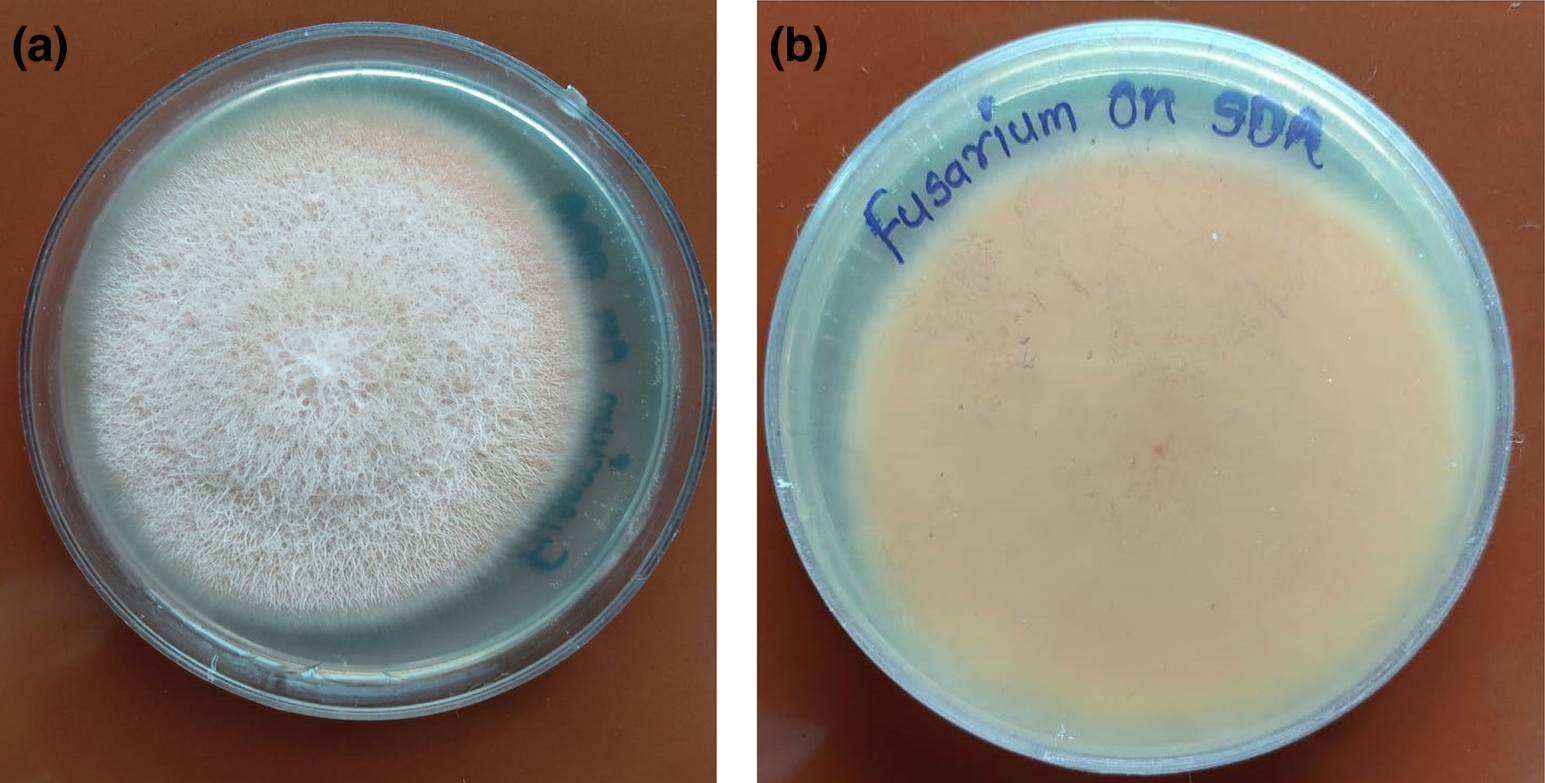
Case 3. *Fusarium solani* growth in (a) obverse and (b) reverse view.

**Fig. 9.**
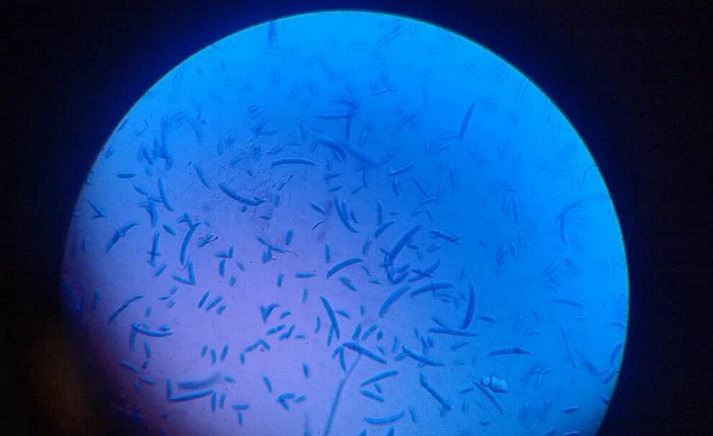
Case 3. LPCB of *Fusarium solani* showing sickle-shaped macroconidia.

On microscopy, micro- and macroconidia were visible. The microconidia were single-celled and in chains or balls. The macroconidia were crescent- as well as bean-shaped and were unicellular and transversely septate. The colonies grew rapidly after 5–6 days, and were granular, flat and floccose. The appearance was white in the early stages, followed by the production of light pink to orange colour. A pigmented *Fusarium solani* was isolated (Figs 8 and 9).

The infection spread to the nasal cavity, orbit area, cerebral area and oral cavity. The doctor recommended FESS and a maxillectomy. She was on ventilator support until her death, and no surgical therapy was performed. She died after 1 month in the ICU following COVID-19 pneumonia, severe lung fibrosis and cardiac shock. Brief information for all three patients is given in Table 1.

**Table 1.** Brief information on patients included in the case series

Case no.	Age (years)/sex	COVID-19 RT-PCR test	Clinical signs/symptoms	Radiological findings	Underlying disease	Vaccinated	Treatment	Species isolated
1	66/M	Positive	Numbness, weakness and tingling on the left side of the face with redness and dropsy of right eye	CT PNS shows an ill-defined heterogenous soft tissue signal intensity and mucosal thickening in maxillary and ethmoidal nasal sinus	Diabetes type 2, hypertension	No	Liposomal amphotericin B	*Neurospora* species
2	63/M	Positive	Respiratory distress, severe headache, facial pain	CT PNS findings were mucosal thickening in maxillary and ethmoidal nasal sinus	Diabetes type 2	No	Liposomal amphotericin B	*Cladosporium* species
3	50 /F	Positive	Respiratory distress, rhinorrhoea, monolateral facial pain	CT PNS and brain showed mucosal thickening in maxillary and ethmoidal sinus	Diabetes type 2	No	Liposomal amphotericin B	*Fusarium* species

*CT PNS: CT scan of paranasal sinus.

## Discussion

Comorbid factors such as diabetes mellitus, immunosuppressive medication, past pulmonary pathology and protracted hospital stay with systemic immunological modifications caused by COVID-19 infection may result in secondary infections, increasing morbidity and mortality.

Intravenous methylprednisolone 0.5–1 mg kg^–1^ day^–1^ for 3 days in moderate cases and 1–2 mg kg^–1^ day^–1^ in severe cases [9, 10] and the use of dexamethasone (6 mg day^–1^ for a maximum of 10 days) in patients who are ventilated or require supplemental oxygen but not in milder cases is recommended by The National Institute of Health [10]. The guidelines specifically mention the risk of developing a secondary infection.

*Zygomycosis* and *Aspergillosis* are the commonest causes of CNS mycosis. Other fungi such as *Candida, Cryptococcus, Coccidioides, Histoplasma* and *Cladosporium* have also been incriminated [11]. Many fungi have probably evolved to switch their lifestyles among endophyte–pathogen–saprotroph. Moreover, when their balanced interaction with the host is disrupted, *Neurospora* species can switch to a pathogenic state. [12]

Our first case reported *Neurospora*, an Ascomycete fungus. Considered a contaminant and commonly utilized in numerous eukaryotic cell biology investigations, it has been implicated in peritonitis, eye infection, oral cavity infection and occupational asthma [13]. In COVID-19 patients, prominent risk factors include incorrect steroid usage and, most critically, diabetes [14] which has an increased likelihood of fungal co-infection with *Neurospora* species, normally considered non-pathogenic to humans.

In our second case, we isolated *Cladosporium* species; this genus comprises dematiaceous saprophytic fungi found in the soil, often referred to as phaeoids [15]. It is an Ascomycete fungus with dark-coloured hyphae due to the presence of melanin, which is regarded as the most significant virulence factor since it interacts with microglial cells. Phaehyphomycosis by *Cladosporium* species in humans is relatively rare, with a variety of clinical presentations including cutaneous, subcutaneous, paranasal sinus and brain infections. Because of their neurotropism, phaeoid fungi are responsible for CNS infection.

Very few cases of ROCM have been reported from Africa, South America and Australia, and the majority are from North America, Asia and the Middle East [13]. According to Katarzyna Góralska *et al*. most patients were farmers by occupation, confirming the primary source of infection, namely soil. Worldwide, males are three times more likely than females to be infected, while a much higher ratio of 14 : 1 is found in India [14, 15]. In our third case we isolated *Fusarium solani* which is saprophytic and widespread in nature [16].

*Fusarium* species are a common worldwide cause of mycotic keratitis, but in immunocompromised patients they may cause various diseases such as onychomycosis, mycetoma, sinusitis and septic arthritis [13]. The main problem of *Fusarium* species is high MICs for amphotericin B. Good clinical and laboratory microbiological practice is thus important, with macroscopic, microscopic and clinical correlation with history for pathogen isolation [17].

ROCM due to such rare species has been sparsely reported in the medical literature. Many post-COVID mucormycosis patients require life-saving FESS and maxillectomy but due to the invasive and aggressive nature of these infections, very few patients survived; those that do survive have permanent deformities affecting their quality of life.

## Conclusion

This study reports three cases of fungal co-infection with extremely rare isolates in COVID-19-positive patients. During the COVID-19 pandemic, India was the country most affected by mucormycosis and ROCM, contributing about 44.3 % of the entire cases reported worldwide.

SARS-CoV-2 has some modulatory effects on the immune response in patients, but treatment based on steroids has had a major impact on immunosuppression, as evident in the recent literature. Prolonged hospital stay in ventilated COVID-19 patients also increases the chances of fungal co-infections with poor outcomes and high mortality. So, it is both the infection and the treatment that has opened the door to secondary infections, which cause considerable morbidity and mortality, as is evident in this case series.

It is therefore vital to be aware that in severely affected COVID-19 patients, particularly those with uncontrolled diabetes and fulminant pulmonary disease, early rapid diagnosis, proper planning, careful management and control of COVID-19 can be successful for the prevention of ROCM, the development of which frequently leads to the patient’s death despite the best efforts of the treating clinicians.

## Limitation

In case 1 and 2, we were able to identify only genus level of causative organism because it can not be able to identify to the species level by conventional methods. Also, their species level identification will not change in to treatment and management plan.
